# Single-Cell Sequencing in Normal and Malignant Hematopoiesis

**DOI:** 10.1097/HS9.0000000000000034

**Published:** 2018-03-01

**Authors:** Nicola K. Wilson, Berthold Göttgens

**Affiliations:** Department of Hematology, Cambridge Institute for Medical Research and Wellcome and MRC Cambridge Stem Cell Institute, University of Cambridge, Cambridge, UK.

## Abstract

Hematopoiesis is one of the best studied adult stem-cell systems, with a differentiation hierarchy progressing from immature hematopoietic stem cells to over 10 distinct mature cell types. Recent technological breakthroughs now make it possible to define transcriptional profiles in thousands of individual cells. Facilitated by the wealth of prior data on cell purification and analysis strategies, hematopoiesis has been one of the earliest experimental systems to which many of these new single-cell sequencing technologies have been applied. In this review, the authors focus on recent studies, which have shed light on heterogeneity within individual populations as well as the relationships between populations, and also attempt to characterize the differences between normal and disease/perturbed states.

## Background

Hematopoiesis is one of the best studied adult stem cell (SC) systems, presumably at least in part due to the ease of sample accessibility and the detailed phenotypic and functional characterization that exists for the various immature and mature blood cell types. Hematopoiesis is often thought of as a step-wise process, which begins at the top of a tree-like structure (the hematopoietic tree) with hematopoietic stem cells (HSCs) at the apex, followed by step-wise branching points via a series of defined progenitor stages to the various differentiated and mature cell types.^1^ Each cell type at the individual stages can be characterized by its surface phenotype using fluorescence activated cell sorting (FACS) and functionally according to its output using in vivo and/or in vitro assays.

The prime function of adult hematopoietic stem/progenitor cells (HSPCs) is to maintain homeostasis within the organism and produce a balanced output of all of the required mature blood cells for the lifetime of the organism. This stability is determined by the ability of HSCs to self-renew, differentiate, or remain quiescent, thereby ensuring that the organism will have a constant supply of blood cells, and can respond to system perturbations such as injury and infection.^2^ In the case of leukemia or other serious blood disorders, normal homeostasis becomes dysregulated and the status-quo is lost.^3,4^ Importantly, cell fate choices such as self-renewal and differentiation are made at the level of individual single cells, and yet must be coordinated (most likely by both intrinsic and extrinsic factors) to maintain the overall balance of the system.^5,6^ Of note, the exact structure of the hematopoietic tree is still hotly debated, as is the extent of heterogeneity within cell populations, the exact process of lineage decision making and how these decisions are perturbed in disease.^7^ Single-cell molecular profiling has emerged as a new and powerful experimental tool to advance our understanding of all of these points.

**Figure 1:**
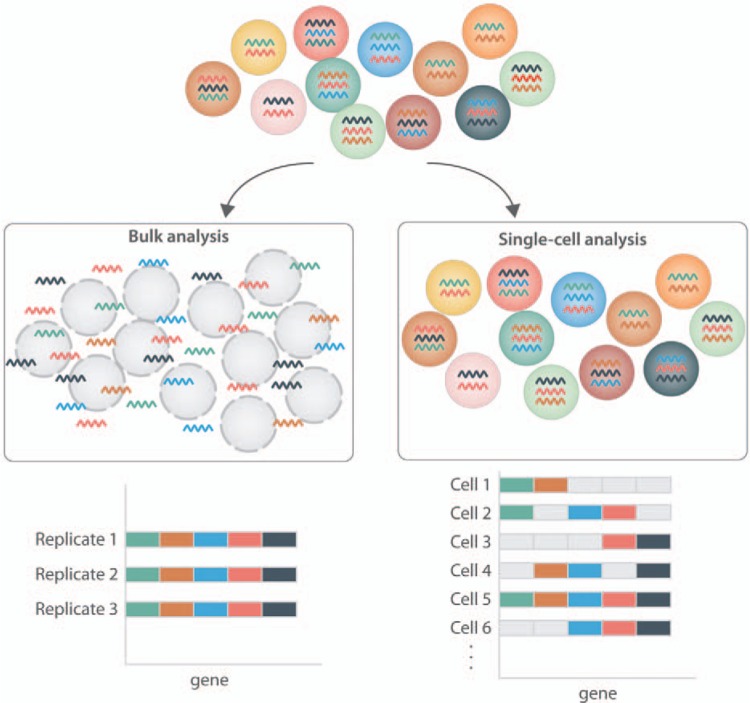
**Cellular heterogeneity can be resolved by single-cell molecular profiling.** Classical bulk gene expression analysis generates population average measurements, which obscure any information about heterogeneity between individual cells. Importantly, cellular heterogeneity is pervasive across many biological settings, including both normal and malignant hematopoiesis. Modern single-cell molecular profiling technologies can resolve cell-to-cell heterogeneity, and thus provide new insights into normal differentiation processes and their underlying regulatory networks, cellular responses to external signals and the heterogeneous cell states present during leukemia development.

Hematopoietic research has long focused on individual single cells. The long-established colony assay, for example, reads out the ability of an individual cell to give rise to a colony of blood cells, and then based on the mature cell types generated, assigns a given progenitor function in this essentially retrospective assay. Similarly, the ultimate gold standard to determine whether a given cell is a HSC is to perform single-cell transplantations and evaluate its ability to reconstitute the blood system of an irradiated recipient.^8–12^ Importantly, hematopoiesis research has a long-track record of pioneering new techniques and single-cell biology is not novel to the twenty-first century. It has long been recognized that bulk RNA-Seq can provide global gene expression where a general overview of a homogeneous population is required, but it will not provide specific information regarding the gene expression changes, which occur on a cell-to-cell basis. This information can be important when trying to look at a heterogeneous cell population and the stochastic processes taking place or the response of a particular cell type to a stimulus (Fig. 1). Single-cell transcriptome analysis of the hematopoietic system was already taking place in 1990, beginning with work in the laboratory of Norman Iscove, which demonstrated that low abundance transcripts could be detected from single cells in a cell-specific manner.^13^ By 1996, Hu et al had been able to adapt real-time polymerized chain reaction (RT-PCR) methods to the single-cell level, and used this approach to highlight the promiscuous nature of multipotent progenitor cells, whereby single multipotent cells expressed multiple “lineage-specific” gene programs proceeding commitment to a specific cell lineage.^14^ This was a landmark paper, which unequivocally showed that the different lineage programs could be detected in one individual cell rather than specific subpopulations of the progenitor compartment.

While the techniques developed in the 1990s enabled measurements of gene expression at the single-cell level, the techniques were laborious, low throughput and not quantitative. The next leap in single-cell technologies came with the use of microfluidics to highly parallelize quantitative RT-PCR (qRT-PCR) from single cells,^15^ and mixing the amplified cDNA with PCR reagents in nanoliter volumes to improve reaction efficiency and save on reagent costs. This technology was subsequently commercialized by the establishment of Fluidigm® and the release of the Fluidigm Biomark™ system, which greatly facilitated expansion of single-cell molecular profiling to a wider range of laboratories. While this technology works very efficiently, the microfluidics approach had several caveats, with 2 of the most influential being the cost and the restriction in terms of the number of handpicked genes which could be analyzed.

To address these shortcomings and analyze more cells and more genes, researchers developed protocols that enabled single-cell RNA sequencing (scRNA-Seq). While the initial protocol was low throughput and expensive,^16^ the Smart-Seq2 protocol published by Picelli et al^17^ allows more efficient mRNA sequencing from a single cell, and can be parallelized either following a plate-based method as in the original protocol or implemented on microfluidic chips developed by Fluidigm (up to 800 cells in 1 experiment). The Smart-Seq2 protocol is of relatively lower cost than any preceding technology and the processing time is comparatively quick. To isolate single cells, complex populations are typically purified further by FACS. If plates are to be used, this also enables the collection of FACS index information, which records precise fluorescence values for each individual cell, and thus provides potentially powerful metadata for subsequent analysis.^18^

For routine analysis of cell numbers that exceed the low hundreds, Smart-Seq2-based protocols, however, remained cost prohibitive. In the quest of alternatives, the largest reduction in cost of scRNA-Seq has come about from protocols such as Drop-Seq and inDrops,^19,20^ which allow a much larger number of cells to be processed bringing down the cost per cell. Briefly, the Drop-Seq protocol is based around encapsulating single cells and the reagents required for cDNA synthesis in a nanoliter droplet using a microfluidic device. The cells are then lyzed within the droplet and cDNA synthesis performed. During cDNA synthesis unique cell barcodes (and unique molecular identifiers [UMIs]) are incorporated into the molecules. This means that each cell has a unique barcode and each transcript from within that cell will contain a UMI. The use of UMIs allows counting of individual transcripts, thereby preventing the same transcript been counted repeatedly. This was a problem with other scRNA-Seq techniques as PCR duplication (the same transcript counted multiple times) was difficult to control. After cDNA synthesis the cells are pooled, amplified, and a sequencing library produced. A commercial provider (10× Genomics®) has now entered the marketplace ensuring more widespread availability and adaption of the technology. The commercial Chromium™ platform from 10× Genomics®, for example, permits the capture of up to 10,000 cells in <6 minutes, and costs for generating sequencing libraries are very cheap on a per-cell basis, because the use of barcodes permits pooling of cDNAs prior to library generation. There are of course caveats with the Drop-Seq/inDrop protocol, as the sequencing data are much shallower when compared with the Smart-Seq2 protocol and cell surface marker information is lost.

**Figure 2:**
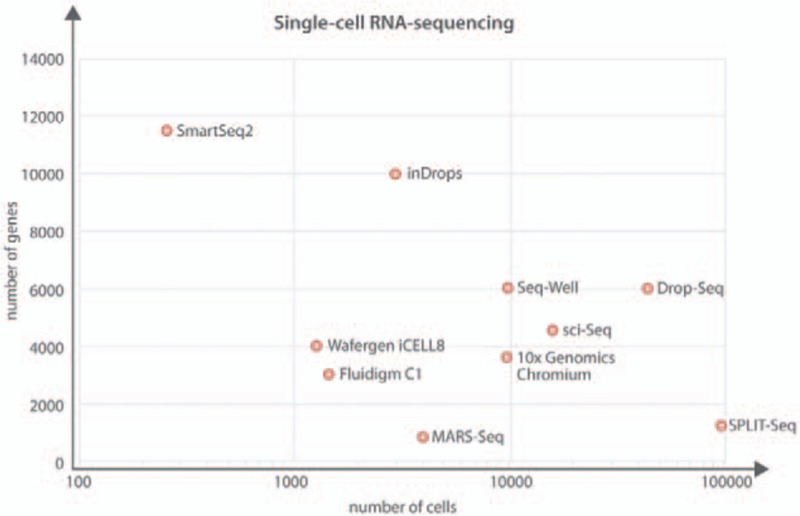
**Summary of single-cell RNA sequencing methods discussed in this review.** Representation of how evolving technologies are permitting the scaling of cell and transcript/gene number. Source references.^17,19,20,23,24,31,61,62^ An average has been taken for genes/transcripts when a range was given in the original text.

For a researcher needing to make a choice about which technology to use, this commonly becomes a balance between cell number, read depth, and cost.^21^ Of note, new protocols reported recently allow the simultaneous capture of epitope and transcriptome,^22^ which now opens up the opportunity of recording specific cell surface markers as part of droplet-based scRNA-Seq protocols. Other protocols recently reported include Seq-Well,^23^ which takes advantage of the Drop-Seq processing method but has no requirement for the capture of cells in droplets. Instead, single cells are captured into wells of a chip using gravity, relying on the fact that the well size will only permit one bead and an individual cell. Importantly, this allows visual inspection of cells, and in due course may be adapted to high content multichannel cell imaging. The commercial Wafergen ICELL8 platform follows a similar concept, and allows for the isolation and processing of 1800 single cells which are all verified visually, so that only wells in which cells meet the predefined criteria are processed further. When choosing which technique to use for an experiment it is important to weigh the benefits against the caveats of each technique. While Smart-Seq2 gives a greater coverage of genes it is lower throughput and more expensive per cell when compared with Drop-Seq (or equivalent). Drop-Seq and 10× Chromium™ on the other hand can process many more cells but the gene coverage is much shallower meaning that it may be difficult to characterize cells, which have a low transcriptional rate or are quiescent (Fig. 2).

One of the main advantages of the ultra-high throughput protocols (Drop-Seq, 10× Chromium™, Seq-Well, and ICELL8) is the inclusion of UMIs. Using combinations of cell barcodes, UMIs, and sequencing library indexes, thousands of individual cells can be pooled together reducing the number of samples to be processed but also ensuring that transcripts are only counted once (to avoid artifacts from PCR duplicates). Much of the Drop-Seq/inDrop data when compared with Smart-Seq2 data would appear as binary data, with genes either detected once or not at all. One of the most likely underlying reasons is that UMI-based methods only count the very 3′ end of each transcript, whereas the Smart-Seq2 protocol can generate sequence reads along the entire reverse transcribed cDNA. A recent development in the single-cell field is that of split barcodes, which enables the processing of large numbers of cells by taking advantage of combinatorial indexing methods. While single-cell combinatorial indexing RNA sequencing (sci-seq)^24^ incorporates different indexes at the mRNA and PCR stages, Split Pool Ligation-based Transcriptome sequencing (SPLiT-seq)^25^ uses in situ ligation to link a well-specific DNA barcode onto the 5′ end of the cDNA molecule as well as incorporating UMIs to allow molecule counting. This process of repeat barcoding ensures that the probability of 2 cells containing the same cell barcode is very low, and that detection of doublets will be easier upon demultiplexing of the samples. These ground-breaking techniques have the potential to revolutionize scRNA-Seq as they will allow the analysis of thousands of cells in a single experiment and drastically reduce the price of processing. The most important consideration when making a choice about which technology to use remains that this needs to be driven by the scientific question. One further issue is that with ever larger cell numbers, the challenge of interpreting the data grows substantially.^26^

## Advancing our understanding of nonmalignant hematopoiesis by single-cell sequencing

The hematopoietic research field was an early adopter of scRNA-Seq technology, which has now been used to investigate a number of different hematopoietic cell types from both human and mouse. While this review focuses on adult hematopoiesis, a substantial amount of research is also focusing on developmental hematopoiesis,^27^ with single-cell approaches relevant not just because of the limiting cell numbers, but also because it is increasingly recognized that there is substantial cellular heterogeneity right from the earliest stages of blood development.^28^ Of note, some early applications of single-cell expression profiling to embryonic hematopoiesis are already making a large impact,^29^ highlighting the first whole transcriptome analysis of early mesoderm formation,^30^ implicating previously unrecognized pathways in tissue development^31^ as well as shedding light on the specific roles of individual transcription factors (TFs) during development.^30,32^ For adult hematopoiesis, different approaches have been taken when isolating cells, from a more general sorting strategy which captures multiple cell types (eg, HSCs and progenitors [Lin^−^Sca1^+^c-Kit^+^], myeloid progenitors [Lin^−^c-Kit^+^Sca1^−^]) to more restrictive strategies which specifically isolate individual cell populations (eg, granulocyte monocyte progenitors [GMP], common myeloid progenitors [CMP], and specific subtypes of dendritic cells). Some of the first studies took advantage of scRNA-Seq and indexomics (FACS index sorting), for example, to refine cell sorting strategies for HSCs, which resulted in a purer population which had over 65% transplantation efficiency when assayed by single-cell transplantation/long-term reconstitution experiments.^33^ A second study went further, and molecularly profiled dormant HSCs.^34^ This study showed that there was a gradual molecular progression toward activation from dormant HSCs to active HSCs, and highlighted specific transcriptional programs which were involved at each progressive step. Other studies have analyzed the differences between young and aged HSCs, showing that the one of the largest varying factors is cell cycle,^35,36^ as well as increased myeloid gene expression.^36^ A further study focused on aged progenitor cells (LMPP/MPP4) and showed that in addition to cell cycle effects in the progenitor cells, there was also a reduction in lymphoid priming genes.^36^ A study investigating the effect of stress (bleeding) on HSPCs showed that induced anemia causes cell cycle changes in both HSCs and MPP1 cells,^37^ yet expression changes were more nuanced with HSCs upregulating Sca1 expression and immune response genes whereas MPP1 cells upregulated granulocytic, megakaryocytic, and erythroid genes. Of note, this study suggests that there may be a link between cell cycle stage and the ability to respond to external stimuli, which would impact the self-renewal/differentiation decision-making processes of many progenitor cell types, and be particularly relevant for quiescent HSCs.

An important focus to date within normal hematopoiesis research has been to delineate the extent of heterogeneity within classically defined hematopoietic cell populations. An important consideration here is the need to determine the difference between the generic heterogeneity (driven by processes occurring across all cell types such as cell cycle) and informative heterogeneity which is driving differences in functional outcome between cell populations. A second major focus has been to identify the genes, which are responsible for driving lineage determination. A prime example of this can be found in a landmark study by the Amit lab, in which they set out to characterize the transcriptional diversity of myeloid progenitors.^38^ Importantly, this study took an unbiased approach to map the transcriptional landscape of myeloid progenitors by performing scRNA-Seq (MARS-Seq^39^) on over 2700 single cells (lin^−^ c-Kit^+^ Sca1^−^). Bioinformatic data analysis was able to separate the single cells into multiple transcriptionally distinct clusters, which recapitulated known gene expression patterns as well as implicate TFs never before associated with specific lineages. The study also looked into the transcriptional effects of perturbations of the hematopoietic system, and demonstrated that although specific TFs where associated with specific lineages, perturbation lead to different effects on the different trajectories. Loss of Cebpα resulted in a complete loss of specific clusters associated with myeloid subgroups whereas loss of Cebpε lead to an increase of neutrophil gene clusters. This study highlights the importance of functional studies to test the hypothesis highlighted by transcriptome analysis.

A comprehensive single-cell transcriptomic survey of the stem and progenitor compartment was provided by a resource paper, which profiled more than 1600 HSPCs using the Smart-Seq2 method,^40^ and provides a web interface that allows investigators to query the expression of any gene of choice (http://blood.stemcells.cam.ac.uk/single_cell_atlas.html). The aim of this study was to interrogate the heterogeneity and profile the higher tiers of the hematopoietic tree at single-cell resolution to recapitulate the earliest stages of the hematopoietic hierarchy at single-cell resolution. The use of broad sorting gates coupled with comprehensive FACS index information also facilitates the investigation of dynamic processes which may take place during specific lineage decisions, both at the cell surface marker expression (using index data) and the transcriptomic level. As illustrated by the authors,^40^ generating reference datasets for normal HSPCs also permits the projection of additional scRNA-Seq datasets, thus enabling comparison to and interpretation of perturbed systems, which is then entirely driven by the underlying molecular expression differences rather than having to rely on external data sources such as gene ontology databases.

Comprehensive scRNA-Seq has also been used to dissect the heterogeneity of mouse GMPs. To determine the most informative genes for this type of analysis, Olsson et al developed a new iterative clustering and guide-gene selection (ICGS) algorithm,^41^ which uses pair-wise correlations of dynamically expressed genes and iterative rounds of clustering to determine the most influential genes within a given dataset. scRNA-Seq was performed on defined hematopoietic populations (HSPCs [LSKs, Lin^−^Sca1^+^c-Kit^+^], CMPs, GMPs, and LKCD34^+^ cells [lin^−^c-Kit^+^CD34^+^]). ICGS analysis was then able to recapitulate the hematopoietic hierarchy but more interestingly was also able to subdivide the GMPs into 3 distinct regulatory states defined by TF–gene pairs. Reassuringly, this included known interactions (Irf8–Klf4), but also identified previously unknown regulatory interactions between specific TFs, such as Irf8 and Zeb2 as well as reciprocal expression patterns between Irf8 and Gfi1. What sets this study apart is that the authors then used this information to capture the rare cells that undergo monocytes to neutrophil specification based on low-level expression of antagonizing TFs. This is an illustration, therefore, how step-wise analysis allows us to understand more of the complex processes involved in the multistep process of lineage determination. Moreover, this paper serves as an example highlighting the need for functional follow-up to validate hypotheses generated by single-cell transcriptomic studies.

**Figure 3:**
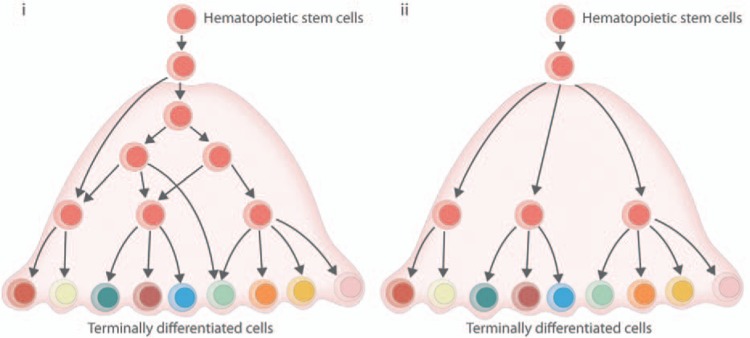
**The hematopoietic hierarchy in the era of single-cell gene expression analysis.** The exact structure of the hematopoietic tree is still hotly debated. (i) A representation of the classical hematopoietic tree, where cells advance in a step-wise progression of differentiation and maturation, from HSCs to mature cell types. (ii) Representation of a more continuous model of differentiation based on recent studies suggesting that hematopoietic stem/progenitor cells cannot easily be resolved into specific HSC and progenitor populations. HSC, hematopoietic stem cell.^42,43^

Recent studies aimed to profile the heterogeneity of the human hematopoietic stem and progenitor compartment, and concluded that it was not possible to resolve the HSPCs into HSCs and oligopotent progenitors.^42–44^ One approach taken used Microwell-Seq^43^ to profile more than 50,000 human hematopoietic cells from GCSF mobilized peripheral blood donors, which recapitulated the multiple different lineages of the hematopoietic system using the Seurat analysis package.^45^ The second study isolated HSPCs (Lin^−^CD34^+^CD38^−^) and committed progenitors (Lin^−^CD34^+^CD38^+^) from healthy bone marrow and performed scRNA-Seq.^42^ In an attempt to define the transition during lineage commitment from SCs to committed progenitors, Velten et al^42^ developed a computational tool (STEMNET) to reconstruct differentiation trajectories. The STEMNET algorithm uses genes specific to the committed progenitors to try to determine the lineage priming of a HSPC. This algorithm is efficient at separating the more committed and intermediate progenitors, but failed to identify clear subgroups within the most immature compartment of the HSPCs. Coupled with comprehensive single-cell culture assays, this lead the authors to conclude that adult human HSPCs exist in a continuum of lowly primed expression states, that rapidly progress to unilineage progenitors with little discernible multipotent progenitor states. This idea therefore challenged more conventional models where the process of lineage specification was thought to be mediated by the step-wise progression of expressing sequential gene modules from multi- to oligo- and ultimately unipotent progenitors.^46^ It will be interesting to see if oligopotent progenitors can be identified within the HSPC compartment using different analysis techniques which do not rely on gene sets defined by already committed progenitors (Fig. 3).

In a more directed approach, Villani et al^47^ set out to characterize subtypes of human dendritic cells (Lin^−^HLA-DR^+^CD14^−^) and monocytes (Lin^−^CD14^lo/++^). Unsupervised analysis of the dataset made it possible to identify combinations of specific cell surface markers, which permitted the isolation of purer dendritic subtypes. Interestingly, although overlapping expression profiles of the 2 cell types could be seen, a common precursor could not be identified. Monocytes were found to be more heterogeneous than previously thought and in addition, two previously unidentified subtypes were seen. Importantly, this study not only identified new subtypes of dendritic cells based on molecular profiles but then functionally supported this by isolating a new subtype from multiple individuals followed by visualization in situ within the tissue. The authors could also isolate a more primitive progenitor cell and compare these cells to previously published studies. While alone this study does not provide an immune atlas it provides a useful resource and a more comprehensive understanding of the role of dendritic cells in tissues, inflammation, and disease.

## Advancing our understanding of malignant hematopoiesis by single-cell sequencing

In the study of malignant hematopoiesis, much focus has been placed on determining the clonogenicity, and this work has investigated the heterogeneity at the level of the transcriptome, epigenome, and resistance of clones to therapy.^48–50^ Many efforts have also been placed on determining not only the driving mutations, which cause leukemia but also the order of mutations and the arrangement of the perturbed hematopoietic tree in disease. The focus of this section of the review will be the transcriptomes of malignant hematopoiesis and the novel insights learned from using single-cell profiling approaches.

An important aspect when studying blood cancers is that both normal and malignant blood cells will be present within a given patient sample. Each individual patient will have a different disease burden which to date has been a technical caveat of single-cell technologies, because genotyping individual cells with low false-negative rates represent a formidable challenge. It is self-evident that knowledge of the mutational status of any given single cell represents crucial information when analyzing its transcriptome. Detection of mutation within the mRNA by scRNA-Seq is heavily dependent on the location of the mutation, because many of the current scRNA-Seq protocols are 3′ biased. Consequently, mutations which are a considerable distance from the polyA site or are in a region which is difficult to amplify (eg, GC rich) cannot be reliably detected. Recently, Giustacchini et al^51^ published a modification to the Smart-Seq2 protocol, which includes specific primers for the site of the mutation (breakpoint region) within the BCR-ABL oncogenic fusion gene. This allowed the detection of the mutant mRNA with a high degree of accuracy, and thus enabled the separation of human HSPCs from chronic myeloid leukemia (CML) patients into BCR-ABL^+^ and BCR-ABL^−^. Using a combination of single-cell transcriptome profiling, mutation characterization, and cell surface phenotype, the investigators were then able to determine the heterogeneity at play within the cancer SCs. The authors identified multiple gene sets, which were specifically enriched in the BCR-ABL^+^ SC but could also detect genes, which were able to differentiate BCR-ABL^−^ HSC in CML patients from normal HSCs in control samples. By being able to sample at diagnosis and then after treatment, the study could also retrospectively predict how patients would respond to treatment from their gene expression profile, and see at diagnosis an enrichment of genes associated with increased proliferation (MYC, E2F, and G2M-checkpoint genes) for good responders and a more quiescent gene profile from poor responding patients. This type of analysis highlights the importance of determining the mutational status of individual cells from patients as well as sampling at diagnosis and after treatment to have a greater predictive power for future treatment regimes and pave the way for a more personalized form of treatment.

To investigate the cooperative nature of Flt3 and Dnmt3a mutations in leukemogenesis, Meyer et al^52^ crossed the mouse lines to a myeloproliferative neoplasm model (Flt3^ITD/ITD^; Dnmt3a^fl/fl^) and acute myeloid leukemia (AML) model (Flt3^ITD/ITD^; Dnmt3a^fl/fl^ MxCre). To characterize the AML model, scRNA-Seq was performed and ICGS was run to identify potential biomarkers, which could be used to access the clonogenic properties of the AML. Expression of IL18ra and c-Myc was shown to be significantly enriched in cells with high clonogenic potential, highlighting the possibility of IL18ra being a surface marker for rare leukemic SCs. The study also highlighted that differentially methylated regions associated with HSPC genes could be reverted upon Dnmt3a rescue, highlighting a potential therapeutic avenue.

## Future directions/conclusions

Hematopoiesis has been at the forefront of single-cell technologies, and the field is increasingly taking advantage of these technologies to address long-standing questions of broad relevance. With experimental protocols maturing and datasets getting ever larger, a major challenge going forward will be the interpretation of these datasets. This includes the immediate issues of the need for large data storage and computer processing power, but also extends to questions of standardization and quality control, which will be essential to perform meaningful cross interrogation between datasets generated in different laboratories. Based on the early successes already reported, it is likely that delving into the datasets will shed light and new perspectives onto which factors, signaling molecules and cascades contribute to the functional heterogeneity of conventionally defined hematopoietic cell populations, what processes drive lineage commitment, and how lineage plasticity can be predicted or altered. In addition to profiling hematopoietic cells, it will also be important to investigate the cells of the hematopoietic niche, not only to characterize potential heterogeneity among different cells that make up HSPC niches, but also to reveal potential cross-talk between HSPCs and their respective niche cells.^53^ Incorporation of additional layers of data may also be of use, for example, single-cell chromatin accessibility or methylome data.^54^ Importantly, work on normal hematopoiesis will provide the foundation for application of single-cell technology to disease states and pertubations, with the real hope of identifying new therapeutic targets that would not be revealed by conventional approaches.

While this would have been thought of as fanciful only a few years ago, the production of large datasets is no longer the limiting factor, while the downstream bioinformatic analysis, intellectual time and thought invested into both the initial design and subsequent interpretation of such vast datasets is now the bottleneck of these types of experiments. The bioinformatic analysis of scRNA-Seq data is comprehensively covered in several reviews, which cover many aspects from the gene-level analysis to cell-level analysis.^26,55^ Mixing the correct blend of investigators will be important as functional studies are needed to complement the transcriptomics to be able to functionally validate any significant findings. Creating knockouts or knock-ins was such a laborious task several years ago but with recent developments in CRISPR-Cas9 technologies reasonably high-throughput testing of multiple novel genes should be attainable.^56,57^ There have also been recent advances made combining CRISPR technologies with scRNA-Seq, such as Perturb-Seq^58,59^ and CROP-Seq.^60^ Approaches such as these once applied to the wider hematopoietic system should begin to shed light on regulatory modules which are playing important roles in hematopoietic development. Finally, building and deepening links with clinicians will also be crucial, because the potential of single-cell profiling for improved diagnosis and/or patient stratification can only be realized if sample collection protocols are adapted so that they are geared to maximize the quality of RNA for subsequent single-cell processing. The future will lie in the interplay of multiple disciplines allowing the further and more in-depth study of single cells. These collaborative efforts will allow us to delve deeper into the regulatory networks and transcriptional programs at play within both normal and malignant hematopoiesis.
